# Longitudinal Analysis of Canine Oral Microbiome Using Whole Genome Sequencing in Aging Companion Dogs

**DOI:** 10.3390/ani13243846

**Published:** 2023-12-14

**Authors:** Ginger B. Templeton, Gilad Fefer, Beth C. Case, Jeff Roach, M. Andrea Azcarate-Peril, Margaret E. Gruen, Benjamin J. Callahan, Natasha J. Olby

**Affiliations:** 1 Department of Clinical Sciences, College of Veterinary Medicine, North Carolina State University, Raleigh, NC 27607, USAmegruen@ncsu.edu (M.E.G.); 2 Department of Medicine, Division of Gastroenterology and Hepatology, and UNC Microbiome Core, Center for Gastrointestinal Biology and Disease, School of Medicine, University of North Carolina, Chapel Hill, NC 27599, USA; jeff_roach@unc.edu (J.R.);; 3 Department of Population Health and Pathobiology, North Carolina State University, Raleigh, NC 27607, USA; bcallah@ncsu.edu; 4 Bioinformatics Research Center, North Carolina State University, Raleigh, NC 27695, USA

**Keywords:** oral, microbiome, canine cognitive dysfunction syndrome, Alzheimer’s disease

## Abstract

**Simple Summary:**

The prevalence of dental disease and cognitive decline in elderly dogs is extremely high, and, given the known relationship between dental disease and Alzheimer’s Disease in people, this study sought to describe the changes in oral microbiota in aged pet dogs over time. By sequencing oral swabs, we were able to identify bacterial and fungal populations in the dogs’ mouths. The most common bacterial species present, *Phorphorymonas* spp. is known to produce factors that cause neurodegeneration. Moreover, *Leptotrichia*, another bacterial species present, correlated to cognition scores in these dogs. We conclude that this small exploratory study shows the importance of defining the oral microbiota in aged dogs with a view to understanding potential therapeutic targets. Larger prospective studies should be undertaken as a priority.

**Abstract:**

Aged companion dogs have a high prevalence of periodontal disease and canine cognitive dysfunction syndrome (CCDS) and the two disorders are correlated. Similarly, periodontal disease and Alzheimer’s Disease are correlated in people. However, little is known about the oral microbiota of aging dogs. The goal of this project was to characterize the longitudinal changes in oral microbiota in aged dogs. Oral swabs were taken from ten senior client-owned dogs on 2–3 occasions spanning 24 months and they underwent whole genome shotgun (WGS) sequencing. Cognitive status was established at each sampling time. A statistically significant increase in alpha diversity for bacterial and fungal species was observed between the first and last study visits. Bacteroidetes and proteobacteria were the most abundant bacterial phyla. *Porphyromonas gulae* was the most abundant bacterial species (11.6% of total reads). The species *Lactobacillus gasseri* had a statistically significant increase in relative abundance with age whereas *Leptotrichia* sp. oral taxon 212 had a statistically significant positive longitudinal association with cognition score. There is an increased fungal and bacterial alpha diversity in aging dogs over time and nearly universal oral dysbiosis. The role of the oral microbiota, particularly *Leptotrichia* and *P. gulae* and *P. gingivalis*, in aging and CCDS warrants further investigation.

## 1. Introduction

The oral microbiota is a complex ecosystem in both humans and dogs [1,2]. Oral-associated bacteria have been implicated in diseases of inflammation and senescence, including cardiovascular disease, diabetes mellitus, cancers, and Alzheimer’s disease (AD) [3,4,5,6,7]. Companion dogs make an excellent model system for healthy and unhealthy human aging as they share the human environment, have diseases in common with humans, and yet, age at an accelerated rate [8]. Defining the oral microbiota of senior and geriatric companion dogs is critical to our understanding of healthy canine aging and senescence, which, in turn, can be applied to a greater understanding of human aging.

The field of canine microbiome research is in its early stages but it is advancing rapidly. A number of studies have focused on the gastrointestinal bacterial microbiome, with fewer investigating the oral and nasal environments. Early studies of oral bacteria were limited by their reliance on culture-based methods. In contrast, recent studies have taken advantage of next-generation sequencing, primarily 16S rRNA amplicon sequencing analysis, to reveal a much greater diversity of organisms in the canine oral cavity. These studies have primarily focused on populations of young to middle-aged dogs and, in many cases, have been limited to purpose-bred, working, or kenneled dogs of single or similar breeds [9,10,11,12]. One recent study reported a diverse fungal population in the canine mouth using internal transcribed spacer analysis [13]. Still, research into the canine oral mycobiome is even less advanced than the bacterial microbiome.

In humans, the progression of periodontal disease correlates with AD progression [14]. Chronic inflammation has been suggested as an indirect cause of this relationship [15], whereas the keystone periodontal pathogen *Porphyromonas gingivalis* has been suggested as a direct cause, mediated by virulence factors called gingipains [16].

Canine cognitive dysfunction syndrome (CCDS) bears remarkable similarity at both the cellular and behavioral levels to AD. Dogs with CCDS develop pathognomonic central nervous system features of AD, including cerebral atrophy, ventricular widening, deposition of plaques of misfolded Aβ in the prefrontal cortex and cerebral vasculature as well as neuronal loss affecting the cerebral cortex, hippocampus, and limbic system [17]. CCDS prevalence has been estimated as high as 67% for dogs aged 16–17 years based on owner-reported symptoms in the domains of disorientation, changes in social interactions, house training and the sleep/wake cycle [18]. Further, findings in our laboratory show a significant decline in performance on laboratory tests of cognition in geriatric companion dogs [19]. This decline is accompanied by escalating serum neurofilament light chain concentrations, and elevations followed by reductions in serum concentrations of amyloid beta 42, mirroring changes seen in patients with Alzheimer’s Disease [18]. Based on these studies of histopathology, MRI findings, blood biomarker alterations, behavioral changes and reduced performance on cognitive tests, companion dogs can serve as an excellent real-world model for AD.

The primary objective of this exploratory study was to define the oral microbiota of senior dogs using whole genome sequencing to enhance species-level accuracy over previous studies relying on 16S rRNA amplicon sequencing. A secondary study aim was to evaluate the correlations between the oral microbiome and naturally occurring age-related disease processes, including CCDS.

## 2. Materials and Methods

### 2.1. Animals

Dogs in this study were companion animals, and owners signed informed consent prior to enrollment in the study. All procedures were reviewed and approved by the North Carolina State University Institutional Animal Care and Use Committee (protocol number 18-109-O) and all work performed was in accordance with the guidelines and regulations. All methods are reported in accordance with ARRIVE guidelines for the reporting of animal experiments.

Senior companion dogs (small dogs > 10 years, large dogs > 8 years, giant dogs > 6 years of age) were recruited for a longitudinal study of neuro-aging. To be included, dogs were required to have intact hearing, intact vision, and the ability to walk independently at the time of enrollment. Dogs with urinary tract infections and those receiving medications that could impair cognition (e.g., antiepileptic drugs such as phenobarbital, potassium bromide, levetiracetam, zonisamide or behavior modifying drugs) were excluded. Subjects were evaluated at approximately 6-month intervals, each evaluation spanning three non-consecutive hospital day-visits. This study spanned the emergence of COVID-19 and, as a result, some assessment dates were delayed or altogether canceled.

To account for differences in life expectancy due to size and breed, lifespan was predicted with the following formula: L = 13.620 + (0.0702H) − (0.0538W) as described [20] where H = height (cm) and W = weight (lbs.). Fraction of lifespan (FoL) was then calculated: FoL = L/age. Both absolute age and FoL were included as independent covariates in statistical analyses.

Table 1 provides information on the dogs recruited into the study.

### 2.2. Covariate Data Collection

At each 6-month evaluation, diet, medications, and supplements being given were recorded and no restrictions were placed. Owners completed the canine dementia scale (CADES) and the canine brief pain inventory (CBPI) questionnaires. The CADES score is a validated metric of neurobehavioral abnormalities exhibited by dogs developing CCDS. The CADES score ranges from 0–95, and subjects are classified as normal (<8), mild (8–23), moderate (24–44), and severe (45–95) [21]. For this study, subjects were divided into two cognitive groups based on CADES scores: normal to mild impairment (NM, 0–23) and moderate to severe (MdSv, 24–95). The CBPI was designed to measure pain and has been validated in the assessment of pain caused by osteoarthritis (OA) [22] and bone cancer [23] and response to pain medication in OA [24].

On day one of each six-month evaluation, one of two veterinarians performed a physical exam, including body and muscle condition score, orthopedic and neurological examination, and indices of gingivitis (GI), plaque (PI), and calculus (CI) as previously described [25]. Oral examinations were not performed under sedation or anesthesia and so detailed dental assessments were not possible.

Oral swabs were collected on the third day of evaluation. A total of 27 samples from 10 dogs were collected on 2–3 dates spanning 23 months. Swabs were performed by one of two veterinarians wearing sterile gloves and using a sterile oral swab. The supragingival and adjacent buccal mucosa were swabbed for 30–60 s using the same swab. No sedation was used when collecting the samples. Samples were immediately placed in sterile tubes on ice and moved to −80 °C within 30 min of collection.

Behavioral and cognitive testing was performed on days 2 and 3 of the evaluation and included inhibitory control (cylinder task, IC), reversal learning (cylinder detour), and the sustained gaze test (SGT) as previously described [19,26].

### 2.3. DNA Isolation and Sequencing

Oral swabs were submitted to the University of North Carolina (UNC) Microbiome Core and treated as follows: DNA isolation was performed using an optimized version of the QIAamp Fast DNA Stool Mini Kit (Cat No./ID: 51604) protocol supplemented with 60 mg/mL lysozyme (Thermo Fisher Scientific, Grand Island, NY, USA) [27,28,29]. DNA was quantified via Quant-iTTM PicoGreenTM dsDNA quantification reagent.

For the validation of the DNA isolation process, a known bacterial community, ZymoBIOMICS Microbial Community Standard (Cat# D6300), and a blank composed of only DNA isolation reagents was included in the DNA extraction process and again in the library preparation. In addition to the isolation controls, the library preparation also included a library blank composed of library preparation reagents alone.

Swift Whole Genome Shotgun (WGS) sequencing was then performed. For samples with a DNA concentration <2 ng/uL, 5 ng of genomic DNA and for samples with DNA concentration >2 ng/uL, 25 ng of genomic DNA was processed using the Swift 2 S Turbo DNA Library Kit (Swift Biosciences, Ann Arbor, MI, USA; Lot#04030036P). Targeted DNA was treated with the Swift 2 S Turbo Enzyme Mix to induce dsDNA fragmentation, repair, and A-tailing. The thermal profile for fragmentation was 32 °C for 11 and 9 min, respectively, based on input concentration, followed by 60 °C for 30 min. Next, the fragmented DNA was ligated using the Swift 2 S Turbo kit ligation mix, followed by a 20 min incubation at 20 °C. Next, index 1(D7) and index 2(D5) were added along with the Swift 2 S Turbo indexing mix. The thermal profile for the amplification had an initial extension denaturation step at 98 °C for 30 s, followed by 8 and 5 cycles of denaturation, respectively, based on input concentration at 98 °C for 10 s each, annealing at 60 °C for 30 s, and a 60 s extension at 68 °C. The DNA library was then purified using Agencourt^®^ AMPure^®^ XP Reagent (Beckman Coulter, Indianapolis, IN, USA). Each sample was quantified and normalized prior to pooling. The DNA library pool was loaded on the Illumina platform reagent cartridge (Illumina, San Diego, CA, USA) and the Illumina instrument [30].

The prepared pool sequencing suitability was initially verified on the Illumina Nano platform. Following successful preliminary sequencing, prepared pools were submitted to the Illumina NovaSeq SP-XP platform with run configuration PE/150x v1.5. Sequencing output from the Illumina NovaSeq platform was converted to the fastq format and demultiplexed using Illumina Bcl2Fastq 2.20.0 [31]. Quality control of the demultiplexed sequencing reads was verified by FastQC 0.11.9 [32]. Adapters were trimmed using Trim Galore 0.6.7 [33]. The resulting paired-end reads were classified with Kraken 2.1.2 [34], and Bracken 2.5 [35] with the addition of the canine and human reference genomes and canine-relevant bacterial species (Supplementary Table S1), and all reads identified as canine or human were eliminated. Bracken output and covariates were loaded into R 4.1.3 [36]. tidyverse 1.3.1 [37] was used for data organization and phyloseq 1.36.0 [38] and vegan 2.6-2 [39] were used for calculation of alpha and beta diversity. Visualization was performed with ggplot2 3.3.5 [40] and JMP Pro 16 [41].

### 2.4. Statistical Analysis

Statistical analysis was performed in JMP Pro 16 [41]. Bacterial and fungal communities were analyzed separately from one another. A change in the Shannon Index (H) or Simpson’s Index of Diversity (1-D) within individuals was analyzed using the Wilcoxon Signed-Rank test for first and last samples of bacterial or fungal. The relationship between bacterial or fungal alpha diversity (H or 1-D) and a number of covariates were analyzed using a linear mixed effects model. Specifically, CADES score or CBPI were treated as the dependent variables, subject as random effect, and FoL or age and alpha diversity metrics as fixed effects, Separately, H or 1-D were treated as dependent variables, subject as random effect, and FoL, age, CADES group (NM, MdSv) and dental prophylaxis (dental cleaning) in the preceding six months or FoL, inhibitory control, and reversal learning as fixed effects.

The abundance of individual taxa was longitudinally analyzed using the negative binomial mixed model (NBMM) [42]. Taxa present in fewer than 20% of samples or assigned fewer than 2500 reads across all samples were removed. In total, 3 models were considered with fixed effects: FoL alone, CADES alone, and FoL and CADES concurrently. In all 3 models sequencing pool was included as a fixed effect and subject was included as a random effect. Analogously, 2 additional models were considered replacing CADES with SGT. Adjusted p-values were reported as calculated by NBMM.

## 3. Results

### 3.1. Clinical Findings

The first 10 dogs enrolled in a larger longitudinal study of canine neuro-aging to provide 2 to 3 consecutive oral (supragingival/buccal mucosal swabs) samples over 24 months were selected for exploratory analysis of the aging canine oral microbiome. At that time, 24 dogs were enrolled in the longitudinal study but many had missed rechecks due to the COVID pandemic. Physical characteristics at the time of enrollment are summarized (Table 1). Subjects were generally in good health throughout the study, though common age-related comorbidities developed and are summarized in Supplementary Table S2 along with owner-reported history of medications, probiotics, and diet.

All owners denied routine toothbrushing or did not respond to the survey question, which was interpreted as not routinely brushing their teeth. Clinical scores of gingivitis, plaque and calculus and history of recent dental prophylaxis (within four months of sample collection) are summarized (Table 2).

**Figure 1:**
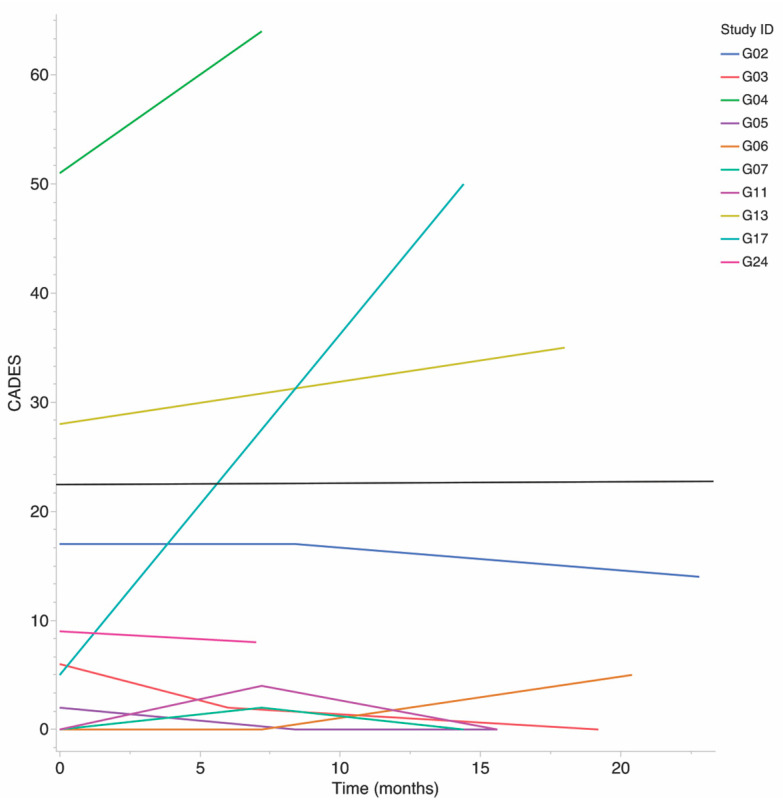
Progression of CADES score over study by subject. Subjects with CADES above 23 (black line) are considered to have moderate to severe (MdSv) cognitive dysfunction. Scores below 23 are considered normal cognition to mild (NM) dysfunction.

One subject experienced severe cognitive decline over the study, as evidenced by a 45-point CADES score increase. The remaining nine subjects were stable within their CADES group throughout the study, seven were classified as NM, and two were classified as MdSv, Figure 1. CADES and CBPI scores were not obtained at the third (last) study visit for subject G04. CADES was in the MdSv group for this subject at the first and second visits and thus was assumed to be MdSv for the third and treated as the last observation carried forward for both CADES score and CADES group.

### 3.2. Microbiome Findings

The datasets generated and analysed during the current study are available in the NCBI Sequence Read Archive repository, https://www.ncbi.nlm.nih.gov/sra/PRJNA950991 (accessed on 28 April 2023) Submission ID: PRJNA950991.

Only 1 sample (Subject G02, Sample 1) was discarded due to low reads (34,243). The remaining 26 samples ranged from seven to 16 million (7,056,183–16,211,066) raw reads.

BRACKEN (Bayesian Reestimation of Abundance with KrakEN) estimates of canine host content ranged by sample from 22.2–99.6%, with a median 92.0% and mean 84.2%. Estimated percent human sequence was less than 0.2% in all samples. Of the remaining taxa, bacterial relative abundance was 99.4%, eukaryotic (fungal) relative abundance was 0.5%, and less than 0.001% was archaeal or viral. Due to low abundance, viral and archaeal taxa were removed prior to subsequent analysis. Fungal and bacterial taxa were analyzed and reported as two separate populations.

**Figure 2:**
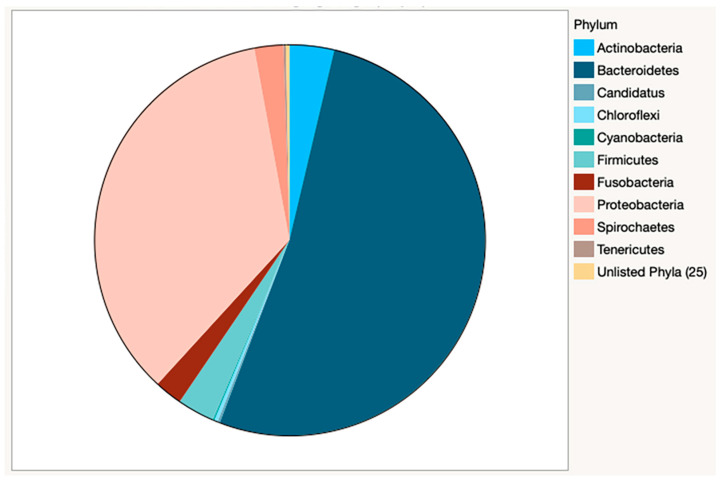
Relative abundance of bacteria by phyla in combined samples. The dominance of Bacteroidetes and Proteobacteria is clearly visible in this pie chart. Actinobacteria and Firmicutes account for nearly 7% of the bacterial phyla present.

Bacterial species counts from Kracken2/Bracken analysis are reported by sample (Supplementary Table S3). 5378 bacterial species were identified from 35 phyla. Of these, 10 phyla accounted for greater than 99.7% of relative abundance (Table 3), with the majority of species classified as Bacteroidetes (52.1%) or Proteobacteria (35.3%) (Figure 2).

**Figure 3:**
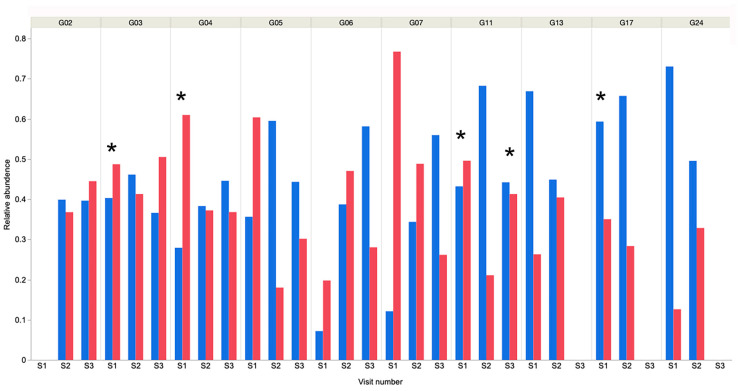
Relative abundance of Bacteroidetes and Proteobacteria in each sample from each dog over time. It is evident that in all but dog G06, S1 these 2 bacterial phyla account for the majority of bacteria present. * Indicates that dental prophylaxis was undertaken within the 6-month period prior to oral sampling. Blue bars represent Bacteroidetes and red represent Proteobacteria.

Actinobacteria (3.7%) and Firmicutes (3.1%) were the third and fourth-most abundant phyla, respectively. These findings were similar to those in a study of young working dogs [12], but in contrast to much of the existing canine oral microbiome literature in young to middle-aged dogs, where the Firmicutes are predominant [9,11] or in much higher abundance [10]. It should be noted that previous reports used 16S rRNA amplicon sequencing analysis and these different methodologies could yield very different results, making direct comparison challenging. Within individual samples, Bacteroidetes or Proteobacteria were the predominant phyla in 25 of 26 samples (Figure 3), and Tenericutes was predominant in the remaining sample.

**Figure 4:**
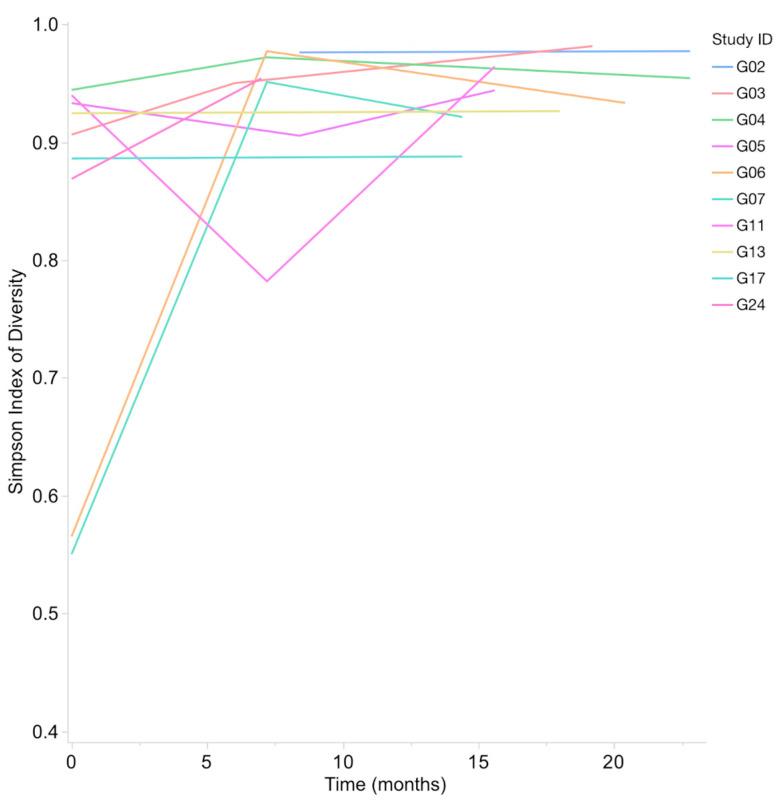
Simpson’s Index of Diversity (1-D) for bacteria increases in all subjects from first to last sample. This increase in bacterial alpha diversity over time was significant (*p* = 0.002).

Shannon Index (H) range for bacterial taxa from oral samples was 1.97–5.57, with a median of 4.15 and mean of 4.18. The Simpson’s Index of Diversity (1-D) range for bacterial taxa was 0.55–0.98, with a median of 0.94 and a mean of 0.90. A statistically significant increase in bacterial alpha diversity was observed between the first and last samples (Wilcoxon Signed-Rank, *p* = 0.0039 for Shannon Index and *p* = 0.002 for Simpson’s Index of Diversity) (Figure 4).

In total, 546 bacterial species, each consisting of greater than 0.01% of total counts, represented 89.7% of the total species estimated. The canine oral pathogen, *Porphyromonas gulae*, was the most prevalent (11.6%), with the closely related human oral pathogen, *P. gingivalis* at 3.1%. These two organisms are considered keystone species in periodontal disease in their respective hosts [43,44] and will be collectively referred to as Gingipains-producing *Porphyromonas species* (GPPs). The second-most abundant species was *P. cangingivalis* (10.5%), which has been reported as the predominant health-associated oral species in adult dogs and is also common in early periodontal disease [45]. In addition to GPPs, the two other “Red Complex” species, *Tannerella forsythia* (3.4%) and *Treponema denticola* (1.6%), known for their role in periodontal disease [14], were among the 20 most abundant species in the study (Table 4).

### 3.3. Relationship between Microbiome, Age, Cognition and Pain

Using a linear mixed effects model, with the subject as a random effect, there was no statistical correlation between bacterial alpha diversity (H or 1-D) and age, FoL, CADES, CADES group, CBPI, SGT (for H), inhibitory control, reversal learning, or recent dental prophylaxis (H and 1-D) (see Supplementary Table S4). There was no significant correlation of bacterial alpha diversity (H or 1-D) with the combined relative abundance of the two known producers of the virulence factor gingipains, *P. gulae* and *P. gingivalis* (heretofore collectively referred to as GPPs) or the combined relative abundance of the so-called “red complex bacteria”, a defined group of human periodontal pathogens consisting of *P. gingivalis, T. forsythia,* and *T. denticola*, and including *P. gulae* in this report [15]. There were no apparent trends in beta diversity (Bray–Curtis) with respect to CADES, age, or FoL (see Supplementary Table S5 for the Bray–Curtis matrix). However, the lack of significant relationships could simply represent the small sample size.

Longitudinal analysis of individual taxa using negative binomial mixed models including FoL and sequencing pool as fixed effects and subject as random effect showed the species *Lactobacillus gasseri* to have a statistically significant (adjusted *p* = 0.0006) increase in relative abundance with FoL. Models with fixed effects CADES and sequencing pool with subject as a random effect indicated a modest, yet statistically significant (adj. *p*-value 2.9289 × 10^−5^) increase in relative abundance of *Leptotrichia* sp. oral taxon 212 with increased CADES score. Models including both FoL and CADES as well as sequencing pool as fixed effects and subject as a random effect again identified increased *Lactobacillus gasseri* as significantly associated with increased FoL. However, for *Leptotrichia* sp. oral taxon 212 neither FoL nor CADES were statistically significant likely due to the correlation between FoL and CADES. Model coefficients and *p*-values for all models are reported in Table 5. No species were identified to be longitudinally associated with SGT in any of the tested models.

### 3.4. Fungal Species

The Shannon Index (H) range for fungal taxa was 0.99–3.74, with a median of 1.47 and a mean of 1.71. The Simpson’s Index of Diversity (1-D) range was 0.36–0.86, with a median of 0.49, and a mean of 0.53. A statistically significant increase in fungal alpha diversity was observed between first and last samples (Wilcoxon Signed-Rank, *p* = 0.07 for Shannon Index and *p* = 0.01 for Simpson’s Index of Diversity) see Supplementary Table S2. Using a linear mixed effects model, with subject as random effect, there were no statistically significant correlations between fungal alpha diversity (H or 1-D) and age, FoL, dental prophylaxis in prior six months, CADES score, CBPI, inhibitory control, reversal learning or SGT.

In total, 66 fungal species, each of which consisted of at least 0.01% of total fungal counts, representing 100% of total fungal species estimated, were identified. The most abundant species in total was *Aspergillus oryzae* (67%), which was present in all 26 samples, and the most abundant species in each sample. *A. oryzae* is considered a non-harmful Aspergillus species used in food production, and, to our knowledge, has not been previously reported in studies of the canine oral mycobiome [46]. The opportunistic pathogen *Candida dubliensis,* was present in all samples, while the closely related pathogenic yeast, *C. albicans*, was present in 22 of 26 samples [47]. *Eremothecium sinecaudum*, a plant pathogen that has been identified as a component of the healthy human lung mycobiome, was present in 24 of 26 of samples [48]. Ten additional fungal species were present in all 26 samples (Table 6), the majority of which have been previously described as plant pathogens [49].

## 4. Discussion

To our knowledge this study represents the first use of WGS sequencing to define the oral microbiota of aging companion dogs, and the first to investigate the oral microbiota in this population longitudinally. We identified a bacterial community consistent with oral dysbiosis in the majority of our subjects over the span of the study. Two closely-related organisms, *P. gulae* and *P. gingivalis,* were predominant. These bacteria are considered keystone species in periodontal disease in dogs [43] and humans [14], respectively. Moreover, *P. gingivalis* has been implicated in AD in humans, either indirectly through chronic inflammation or directly through virulence factors including small proteases called gingipains [44]. *P. gulae* produces gingipains and other virulence factors similar to *P. gingivalis* [50]. A recent study showed a statistically significant correlation between periodontal disease and cognitive decline in dogs [51]. Additionally, in a small study of aged beagles, *P. gulae* DNA and gingipains antigen was isolated from brain tissue, though cognition was not measured, and a gingipains inhibitor reduced clinical signs associated with periodontal disease in that study [52].

Longitudinal analysis of individual taxa allows changes from baseline to be interrogated with respect to age and cognitive state regardless of initial state. This aspect is essential given the potentially vast variation in canine oral microbiome at later stages of life. We identified one organism with a statistically significant positive correlation with CADES score, *Leptotrichia* sp. oral taxon 212, and one species with a statistically significant positive correlation with FoL, *Lactobacillus gasseri*. *Leptotrichia* species are facultative anaerobic Gram-negative bacilli, and are common inhabitants of the human oral microbiota [3] that have been associated with both oral health and disease states in humans [53] and in gingivitis in dogs [11]. *Leptotrichia* species have been associated with AD [54,55] and periodontal disease [53]. In humans. *Leptotrichia wadei*, in particular, has been observed to be significantly enriched in subjects with mild cognitive impairment [54]. However, species of the genus *Leptotrichia* have been associated with both periodontal health and disease suggesting that individual *Leptotrichia* species have “distinct pathogenic potential” [53]. The relationship between CCDS and *Leptotrichia*, *P. gulae* and *P. gingivalis* merits further investigation.

Several Lactobacillus species, *Lactobacillus gasseri* in particular, have been previously identified in dog faeces [56,57] and human breast milk derived *Lactobacillus gasseri* has been studied as a probiotic treatment for obesity in dogs [58]. The study of association of *Lactobacillus gasseri* with aging or cognition outside of model organisms [59], however, remains largely limited to the study of probiotic supplementation [60].

The biological significance of the fungal species identified in this study is unclear, but it is interesting that 12 species were found in all 26 samples, including *A. oryzaeh*, the most abundant species present, and the opportunistic pathogen, *C. dubliniensis*. This is in contrast to published findings of the canine oral mycobiome in a younger study population where none of the observed species occurred in all samples [13]. We report a statistically significant increase in both bacterial and fungal alpha diversity over the course of the study. In humans, oral alpha diversity has been shown to decrease with age [61]. Our results may represent a difference between the oral ecosystem of aged humans and senior companion dogs, differences in behaviors between humans and dogs, the onset of age-related behaviors (such as an increase or decrease in self-grooming) or study-related factors such as sample size or duration of this study.

This study was an exploratory evaluation of the oral microbiota and the small cohort size limits the conclusions that can be drawn. The fact that only one dog progressed into the MdSv CADES group during the course of the study limited the study’s power to examine the relationship between cognitive change and changes in the oral microbiota. With a larger sample size and a longer timeline, we anticipate a larger number of dogs progressing through CADES groups. This would allow examination of differences in the oral microbiome pre-CCDS and after the diagnosis of CCDS within individuals, potentially elucidating a role for the oral microbiome in CCDS development or progression. Moreover, cognitive aging occurs at different rates in different dogs and comparison of the oral microbiota between successful agers, those that develop mild impairment and those that develop severe impairment could also be evaluated [62].

Because these data were generated as part of a broader study of neuro-aging intended to investigate natural aging in companion dogs, subjects with antibiotic use and dental prophylaxis were not excluded. As our study size and duration continue to increase, so too will the power, potentially enabling further understanding of the relationship between dental care, medications, oral microbiome, and cognition. At present, we found no correlation between recent dental prophylaxis and either alpha diversity, relative abundance of GPPs, or relative abundance of red complex bacteria. However, it is important to note that comprehensive oral examinations under anesthesia were not performed in these dogs. In this longitudinal study of aging dogs’ health, the central tenet is to cause no harm to the dogs during data collection. As such, anesthesia and sedation are avoided at the routine 6-monthly assessments. The use of the calculus, gingival and plaque indices allowed the description of some aspects of periodontal disease but should not be considered a complete evaluation of dental and oral health.

The high prevalence of cognitive decline in aging dogs coupled with the abundance of *P. gulae* and *P. gingivalis* in the aging cohort in the present study, leads us to postulate that these species may play a role in CCDS. While it is well-established that periodontal disease is very common in aging dogs [63], the extent to which oral dysbiosis contributes to senescence in dogs has not been fully elucidated. Defining causative organisms in CCDS and elucidating the mechanisms involved will provide potential preventative and therapeutic targets for CCDS.

A limitation and advantage of this study is that the intraoral sites sampled (supragingival and buccal mucosa) is a combination of two distinct niches. This limits our ability to compare findings to prior studies. However, we chose this method for its ability to represent a greater portion of the oral cavity than single-site sampling and thus is likely more representative of the overall health state of the mouth. Moreover, establishing the aging oral microbiome using a method that does not require general anesthesia provides a baseline for future studies of the aging canine oral microbiome that may wish to avoid the risk or expense of anesthesia. Understanding this site is also valuable considering the availability of commercially-available tests of the oral microbiome which rely on owner-administered oral swabs for sample collection.

## 5. Conclusions

Dysbiosis of oral microbiota is the norm in aging dogs, in spite of dental prophylaxis. The high prevalence of red complex bacterial species points to a potential contributing factor in the development of cognitive decline and we conclude that evaluation of this relationship should take priority, given the potential to intervene therapeutically.

As our knowledge of the aging canine oral microbiota evolves, so too will our understanding of healthy aging and age-related disease. The oral microbiota is a potential diagnostic tool and therapeutic target. Our study is unique in its longitudinal design, use of companion, rather than lab-housed or purpose bred dogs, and, most importantly, the focus on senior dogs.

## Figures and Tables

**Table 1:** Physical characteristics of study subjects at time of enrollment in trial. M/N: male neutered; F/S: female spayed.

Study ID	Age (years)	Weight (kg)	Sex	Breed
G02	11.6	22.2	M/N	Border Collie
G03	9.5	33.7	F/S	Labrador Retriever
G04	14.8	9.9	F/S	Corgi, Pembroke
G05	10.8	29.7	M/N	German Shepherd Dog
G06	11.1	10.3	M/N	Jack Russell Terrier
G07	11.4	25.4	F/S	Golden Retriever
G11	10.1	27.6	F/S	Mixed Breed (Labrador Mix)
G13	11.0	11.1	M/N	Mixed Breed (Boxer/Pitbull)
G17	11.8	11.1	M/N	Beagle
G24	9.9	8	M/N	Dachshund

**Table 2:** Dental health at time of sampling. Calculus index (CI), gingival index (GI), and plaque index (PI) were recorded at time of microbiome sampling. (nr, not recorded). For the 4 dogs that underwent dental prophylaxis within 6 months of sample collection, the number of months prior is provided.

Study ID	Sample	Calculus Index (CI)	Gingival Index (GI)	Plaque Index (PI)	Prior Dental Prophylaxis (months)
G02	1	nr	nr	nr	
	2	2	2	3	
	3	2	1	2	
G03	1	1	1	1	3.7
	2	1	1	2	
	3	1	1	1	
G04	1	0	2	1	0.7
	2	nr	nr	nr	
	3	1	1	2	
G05	1	2	1	2	
	2	1	1	1	
	3	1	1	1	
G06	1	2	1	2	
	2	2	1	2	
	3	2	3	1	
G07	1	3	1	3	
	2	2	2	2	
	3	2	2	3	
G11	1	1	0	1	2.4
	2	1	1	1	
	3	1	2	2	1.6
G13	1	1	1	1	
	2	1	2	0	
G17	1	2	2	2	0.5
	2	1	2	1	
G24	1	nr	nr	nr	
	2	1	1	2	

**Table 3:** Relative abundance of bacterial phyla, listed in order of greatest to least total abundance, by sample. Bacteroidetes and Proteobacteria account for more than 70% of phyla present in each sample.

ID	Sample Number	Bacteroidetes	Proteo-bacteria	Firm-icutes	Actino-bacteria	Spiro-chaetes	Tener-icutes	Fuso-bacteria	Candi-datus	Chloro-flexi	Cyano-bacteria	Other Phyla
G02	S2	0.3991	0.3679	0.0986	0.0244	0.0674	0.0004	0.0305	0.0017	0.0019	0.0010	0.0071
	S3	0.3966	0.4451	0.0413	0.0428	0.0381	0.0010	0.0285	0.0015	0.0010	0.0015	0.0026
G03	S1	0.4031	0.4872	0.0196	0.0153	0.0113	0.0004	0.0616	0.0004	0.0000	0.0006	0.0005
	S2	0.4614	0.4130	0.0456	0.0282	0.0194	0.0014	0.0184	0.0022	0.0026	0.0023	0.0054
	S3	0.3662	0.5052	0.0386	0.0318	0.0176	0.0007	0.0275	0.0028	0.0028	0.0017	0.0049
G04	S1	0.2793	0.6099	0.0239	0.0569	0.0065	0.0013	0.0213	0.0003	0.0000	0.0001	0.0003
	S2	0.3831	0.3723	0.0760	0.0377	0.0873	0.0028	0.0188	0.0031	0.0101	0.0023	0.0066
	S3	0.4460	0.3681	0.0439	0.0242	0.0800	0.0031	0.0212	0.0021	0.0056	0.0014	0.0044
G05	S1	0.3565	0.6039	0.0189	0.0029	0.0054	0.0006	0.0018	0.0097	0.0000	0.0001	0.0002
	S2	0.5951	0.1802	0.0934	0.0271	0.0368	0.0057	0.0485	0.0075	0.0014	0.0014	0.0028
	S3	0.4434	0.3016	0.0329	0.1197	0.0655	0.0012	0.0146	0.0012	0.0104	0.0017	0.0079
G06	S1	0.0719	0.1977	0.0194	0.0085	0.0276	0.6693	0.0029	0.0006	0.0000	0.0007	0.0014
	S2	0.3872	0.4705	0.0256	0.0490	0.0410	0.0010	0.0185	0.0030	0.0017	0.0006	0.0020
	S3	0.5816	0.2803	0.0533	0.0168	0.0503	0.0013	0.0099	0.0025	0.0018	0.0007	0.0015
G07	S1	0.1212	0.7674	0.0478	0.0417	0.0109	0.0019	0.0068	0.0000	0.0000	0.0000	0.0023
	S2	0.3438	0.4882	0.0323	0.0321	0.0904	0.0008	0.0089	0.0014	0.0002	0.0009	0.0009
	S3	0.5597	0.2616	0.0350	0.0588	0.0490	0.0018	0.0212	0.0036	0.0048	0.0013	0.0033
G11	S1	0.4322	0.4960	0.0440	0.0094	0.0021	0.0000	0.0005	0.0155	0.0000	0.0000	0.0002
	S2	0.6825	0.2108	0.0328	0.0295	0.0114	0.0004	0.0247	0.0028	0.0031	0.0004	0.0015
	S3	0.4423	0.4129	0.0353	0.0755	0.0069	0.0008	0.0103	0.0041	0.0056	0.0018	0.0045
G13	S1	0.6689	0.2630	0.0216	0.0137	0.0096	0.0025	0.0179	0.0018	0.0001	0.0004	0.0005
	S2	0.4490	0.4046	0.0560	0.0694	0.0018	0.0009	0.0147	0.0006	0.0007	0.0002	0.0021
G17	S1	0.5934	0.3503	0.0094	0.0129	0.0040	0.0011	0.0278	0.0000	0.0001	0.0002	0.0007
	S2	0.6573	0.2835	0.0139	0.0129	0.0004	0.0002	0.0303	0.0001	0.0002	0.0003	0.0009
G24	S1	0.7304	0.1262	0.0760	0.0080	0.0186	0.0024	0.0331	0.0033	0.0005	0.0003	0.0011
	S2	0.4956	0.3285	0.0230	0.1002	0.0214	0.0009	0.0239	0.0013	0.0017	0.0006	0.0029

**Table 4:** Relative abundance of bacterial species. Twenty most abundant oral bacterial species in the combined samples. *Porphorymonas* species are the most prevalent bacteria in these samples.

Species	Relative Abundance
*Porphyromonas gulae*	0.116
*Porphyromonas cangingivalis*	0.105
*Bergeyella zoohelcum*	0.076
*Capnocytophaga canimorsus*	0.054
*Pasteurella multocida*	0.047
*Tannerella forsythia*	0.034
*Capnocytophaga cynodegmi*	0.033
*Porphyromonas gingivalis*	0.030
*Neisseria weaveri*	0.028
*Desulfomicrobium orale*	0.024
*Frederiksenia canicola*	0.021
*Treponema denticola*	0.016
*Conchiformibius steedae*	0.016
*Fusobacterium russii*	0.015
*Neisseria shayeganii*	0.013
*Capnocytophaga* sp. *H2931*	0.013
*Capnocytophaga* sp. *H4358*	0.012
*Campylobacter* sp. *CCUG 57310*	0.010
*Porphyromonas crevioricanis*	0.007
*Pseudomonas aeruginosa*	0.006

**Table 5:** Longitudinal analysis of bacterial species, fraction of lifespan and CADES. Bacterial species identified as statistically significant using the Negative Binomial Mixed Model. FoL: Fraction of Lifespan. CADES; Canine Dementia Scale. Three different models are included, one each for FoL and CADES and a third in which both FoL and CADES were included. *Lactobacillus gasseri* is significantly associated with FoL when considered alone or with CADES in the model, and *Leptotrichia* sp. are significant associated with CADES when modeled without FoL. Statistically significant adjusted *p* values are in **bold**.

		Intercept		FoL		CADES		Pool	
Model	Taxa	Coefficient	adj. *p*-Value	Coefficient	adj. *p*-Value	Coefficient	adj. *p*-Value	Coefficient	adj. *p*-Value
FoL + Pool	*Lactobacillus gasseri*	14.6450	2.4732 × 10^−7^	3.8586	**0.0006**	NA	NA	1.9296	0.5512
CADES + Pool	*Leptotrichia* sp. oral taxon 212	−9.2808	2.6547 × 10^−16^	NA	NA	2.2553	**2.9289 × 10^−5^**	−0.5853	0.3086
FoL + CADES + Pool	*Leptotrichia* sp. oral taxon 212	−10.1695	3.8961 × 10^−14^	−0.2427	0.9283	0.9270	0.3838	−0.6415	0.5627
	*Lactobacillus gasseri*	−15.0134	2.0346 × 10^−6^	3.3870	**0.0001**	1.4296	0.2999	2.2378	0.5627

**Table 6:** Oral fungal species. Twenty most abundant fungal species in total. * Represents species found in all 26 samples.

Species	Relative Abundance
*Aspergillus oryzae* *	0.6699
*Colletotrichum higginsianum* *	0.0964
*Ustilago maydis* *	0.0182
*Botrytis cinerea* *	0.0168
*Eremothecium sinecaudum*	0.0102
*Pochonia chlamydosporia* *	0.0101
*Talaromyces rugulosus* *	0.0081
*Candida dubliniensis* *	0.0080
*Kluyveromyces marxianus* *	0.0078
*Thermothielavioides terrestris* *	0.0076
*Zymoseptoria tritici*	0.0074
*Fusarium verticillioides* *	0.0068
*Neurospora crassa*	0.0067
*Ustilaginoidea virens*	0.0066
*Aspergillus luchuensis* *	0.0065
*Thermothelomyces thermophilus* *	0.0061
*Fusarium pseudograminearum*	0.0057
*Pyricularia oryzae*	0.0056
*Naumovozyma dairenensis*	0.0052
*Drechmeria coniospora*	0.0050

## Data Availability

The datasets generated and analyzed during the current study are openly available in the NCBI Sequence Read Archive repository, https://www.ncbi.nlm.nih.gov/sra/PRJNA950991 (accessed on 28 April 2023) Submission ID: PRJNA950991. All data used for individual analyses are provided in Supplementary Tables S1–S5.

## Supplementary Materials

The following supporting information can be downloaded at: https://www.mdpi.com/article/10.3390/ani13243846/s1, Table S1: Species added to the Bracken database based on their presence and abundance in Humann2 output and potential relevance to the canine microbiome. Table S2: Demographic, oral health, cognitive and Shannon and Simpson indices for each individual study participant. Table S3: Bacterial species counts for each sample. Table S4: Results of linear mixed models examining the relationship of outcome variables with bacterial diversity (H and 1-D) with subject as the random effect. Table S5: Bray–Curtis Matrix for prokaryotic species.

## References

[1] Caselli E., Fabbri C., D’Accolti M., Soffritti I., Bassi C., Mazzacane S., Franchi M. (2020). Defining the oral microbiome by whole-genome sequencing and resistome analysis: The complexity of the healthy picture. BMC Microbiol..

[2] Oh C., Lee K., Cheong Y., Lee S.W., Park S.Y., Song C.S., Choi I.S., Lee J.B. (2015). Comparison of the Oral Microbiomes of Canines and Their Owners Using Next-Generation Sequencing. PLoS ONE.

[3] Willis J.R., Gabaldón T. (2020). The Human Oral Microbiome in Health and Disease: From Sequences to Ecosystems. Microorganisms.

[4] Sureda A., Daglia M., Argüelles Castilla S., Sanadgol N., Fazel Nabavi S., Khan H., Belwal T., Jeandet P., Marchese A., Pistollato F. (2020). Oral microbiota and Alzheimer’s disease: Do all roads lead to Rome?. Pharmacol. Res..

[5] Koren O., Spor A., Felin J., Fåk F., Stombaugh J., Tremaroli V., Behre C.J., Knight R., Fagerberg B., Ley R.E. (2010). Human oral, gut, and plaque microbiota in patients with atherosclerosis. Proc. Natl. Acad. Sci. USA.

[6] Karpiński T.M. (2019). Role of Oral Microbiota in Cancer Development. Microorganisms.

[7] Mager D.L., Haffajee A.D., Devlin P.M., Norris C.M., Posner M.R., Goodson J.M. (2005). The salivary microbiota as a diagnostic indicator of oral cancer: A descriptive, non-randomized study of cancer-free and oral squamous cell carcinoma subjects. J. Transl. Med..

[8] Kaeberlein M., Creevy K.E., Promislow D.E. (2016). The dog aging project: Translational geroscience in companion animals. Mamm. Genome.

[9] Ruparell A., Inui T., Staunton R., Wallis C., Deusch O., Holcombe L.J. (2020). The canine oral microbiome: Variation in bacterial populations across different niches. BMC Microbiol..

[10] Wallis C., Marshall M., Colyer A., O’Flynn C., Deusch O., Harris S. (2015). A longitudinal assessment of changes in bacterial community composition associated with the development of periodontal disease in dogs. Vet. Microbiol..

[11] Davis I.J., Wallis C., Deusch O., Colyer A., Milella L., Loman N., Harris S. (2013). A cross-sectional survey of bacterial species in plaque from client owned dogs with healthy gingiva, gingivitis or mild periodontitis. PLoS ONE.

[12] Isaiah A., Hoffmann A.R., Kelley R., Mundell P., Steiner J.M., Suchodolski J.S. (2017). Characterization of the nasal and oral microbiota of detection dogs. PLoS ONE.

[13] Niemiec B.A., Gawor J., Tang S., Prem A., Krumbeck J.A. (2022). The mycobiome of the oral cavity in healthy dogs and dogs with periodontal disease. Am. J. Vet. Res..

[14] Abbayya K., Puthanakar N.Y., Naduwinmani S., Chidambar Y.S. (2015). Association between Periodontitis and Alzheimer’s Disease. N. Am. J. Med. Sci..

[15] Mohanty R., Asopa S.J., Joseph M.D., Singh B., Rajguru J.P., Saidath K., Sharma U. (2019). Red complex: Polymicrobial conglomerate in oral flora: A review. J. Family Med. Prim. Care.

[16] Dominy S.S., Lynch C., Ermini F., Benedyk M., Marczyk A., Konradi A., Nguyen M., Haditsch U., Raha D., Griffin C. (2019). *Porphyromonas gingivalis* in Alzheimer’s disease brains: Evidence for disease causation and treatment with small-molecule inhibitors. Sci. Adv..

[17] Mihevc S.P., Majdič G. (2019). Canine Cognitive Dysfunction and Alzheimer’s Disease—Two Facets of the Same Disease?. Front. Neurosci..

[18] Neilson J.C., Hart B.L., Cliff K.D., Ruehl W.W. (2001). Prevalence of behavioral changes associated with age-related cognitive impairment in dogs. J. Am. Vet. Med. Assoc..

[19] Fefer G., Panek W.K., Khan M.Z., Singer M., Westermeyer H.D., Mowat F.M., Murdoch D.M., Case B., Olby N.J., Gruen M.E. (2022). Use of Cognitive Testing, Questionnaires, and Plasma Biomarkers to Quantify Cognitive Impairment in an Aging Pet Dog Population. J. Alzs. Dis..

[20] Greer K.A., Canterberry S.C., Murphy K.E. (2007). Statistical analysis regarding the effects of height and weight on lifespan of the domestic dog. Res. Vet. Sci..

[21] Madari A., Farbakova J., Katina S., Smolek T., Novak P., Weissovaa T., Novak M., Zilka N. (2015). Assessment of severity and progression of canine cognitive dysfunction syndrome using the CAnine DEmentia Scale (CADES). Appl. Anim. Behav. Sci..

[22] Brown D.C., Boston R.C., Coyne J.C., Farrar J.T. (2007). Development and psychometric testing of an instrument designed to measure chronic pain in dogs with osteoarthritis. Am. J. Vet. Res..

[23] Brown D.C., Boston R., Coyne J.C., Farrar J.T. (2009). A novel approach to the use of animals in studies of pain: Validation of the canine brief pain inventory in canine bone cancer. Pain Med..

[24] Brown D.C., Boston R.C., Coyne J.C., Farrar J.T. (2008). Ability of the canine brief pain inventory to detect response to treatment in dogs with osteoarthritis. J. Am. Vet. Med. Assoc..

[25] Caiafa A. (2013). Oral Examination/Dental Charting and Diagnostic Tools. World Small Animal Veterinary Association World Congress Proceedings.

[26] Bray E.E., Gruen M.E., Gnanadesikan G.E., Horschler D.J., Levy K.M., Kennedy B.S., Hare B.A., MacLean E.L. (2020). Cognitive characteristics of 8- to 10-week-old assistance dog puppies. Anim. Behav..

[27] Allali I., Arnold J.W., Roach J., Cadenas M.B., Butz N., Hassan H.M., Koci M., Ballou A., Mendoza M., Ali R. (2017). A Comparison of Sequencing Platforms and Bioinformatics Pipelines for Compositional Analysis of the Gut Microbiome. BMC Microbiol..

[28] Guadamuro L., Azcárate-Peril M.A., Tojo R., Mayo B., Delgado S. (2019). Use of high throughput amplicon sequencing and ethidium monoazide dye to track microbiota changes in an equol-producing menopausal woman receiving a long-term isoflavones treatment. AIMS Microbiol..

[29] Azcarate-Peril M.A., Butz N., Cadenas M.B., Koci M., Ballou A., Mendoza M., Ali R., Hassan H. (2018). An Attenuated Salmonella enterica Serovar Typhimurium Strain and Galacto-Oligosaccharides Accelerate Clearance of Salmonella Infections in Poultry through Modifications to the Gut Microbiome. Appl. Environ. Microbiol..

[30] Seth-Smith H.M.B., Bonfiglio F., Cuénod A., Reist J., Egli A., Wüthrich D. (2019). Evaluation of Rapid Library Preparation Protocols for Whole Genome Sequencing Based Outbreak Investigation. Front. Public Health.

[31] (2018). Bcl2Fastq.

[32] (2014). FastQC.

[33] (2017). Trim Galore.

[34] Wood D.E., Lu J., Langmead B. (2019). Improved metagenomic analysis with Kraken 2. Genome Biol..

[35] Lu J., Breitwieser F.P., Thielen P., Salzberg S.L. (2017). Bracken: Estimating species abundance in metagenomics data. PeerJ Comput. Sci..

[36] R Core Team (2021). R: A language and environment for statistical computing. R Foundation for Statistical Computing, Vienna, Austria. https://www.R-project.org/.

[37] Wickham H., Averick M., Bryan J., Chang W., McGowan L.D., François R., Grolemund G., Hayes A., Henry L., Hester J. (2019). Welcome to the Tidyverse. J. Open Source Softw..

[38] McMurdie P.J., Holmes S. (2013). phyloseq: An R package for reproducible interactive analysis and graphics of microbiome census data. PLoS ONE.

[39] Oksanen J., Simpson G., Blanchet F., Kindt R., Legendre P., Minchin P., O’Hara R., Solymos P., Stevens M., Szoecs E. (2022). _vegan: Community Ecology Package_. R package version 2.6-2. https://CRAN.R-project.org/package=vegan.

[40] Wickham H. (2016). ggplot2: Elegant Graphics for Data Analysis.

[41] (1989–2021). JMP^®^.

[42] Xinyan Z., Yu-Fang P., Lei Z., Boyi G., Pendegraft A.H., Wenzhuo Z., Nengjun Y. (2018). Negative Binomial Mixed Models for Analyzing Longitudinal Microbiome Data. Front. Microbiol..

[43] Senhorinho G.N., Nakano V., Liu C., Song Y., Finegold S.M., Avila-Campos M.J. (2011). Detection of *Porphyromonas gulae* from subgingival biofilms of dogs with and without periodontitis. Anaerobe.

[44] Fiorillo L., Cervino G., Laino L., D’Amico C., Mauceri R., Tozum T.F., Gaeta M., Cicciù M. (2019). *Porphyromonas gingivalis*, Periodontal and Systemic Implications: A Systematic Review. Dent. J..

[45] O’Flynn C., Deusch O., Darling A.E., Eisen J.A., Wallis C., Davis I.J., Harris S.J. (2015). Comparative Genomics of the Genus *Porphyromonas* Identifies Adaptations for Heme Synthesis within the Prevalent Canine Oral Species *Porphyromonas cangingivalis*. Genome Biol. Evol..

[46] Feng-Jie J., Shuang H., Bao-Teng W., Long J. (2021). Advances in Genetic Engineering Technology and Its Application in the Industrial Fungus Aspergillus oryzae. Front. Microbiol..

[47] Moran G.P., Coleman D.C., Sullivan D.J. (2012). Candida albicans versus Candida dubliniensis: Why Is C. albicans More Pathogenic?. Int. J. Microbiol..

[48] Nguyen L.D., Viscogliosi E., Delhaes L. (2015). The lung mycobiome: An emerging field of the human respiratory microbiome. Front. Microbiol..

[49] Dean R., Van Kan J.A.L., Pretorius Z.A., Hammons-Kosack K.E., Di Pietro A., Spanu P.D., Rudd J.J., Dickman M., Kahmann R., Ellis J. (2012). The Top 10 fungal pathogens in molecular plant pathology. Molec. Plant Patho..

[50] Lenzo J.C., O’Brien-Simpson N.M., Orth R.K., Mitchell H.L., Dashper S.G., Reynolds E.C. (2016). Porphyromonas gulae Has Virulence and Immunological Characteristics Similar to Those of the Human Periodontal Pathogen Porphyromonas gingivalis. Infect Immun..

[51] Dewey C.W., Rishniw M. (2021). Periodontal disease is associated with cognitive dysfunction in aging dogs: A blinded prospective comparison of visual periodontal and cognitive questionnaire scores. Open Vet. J..

[52] Arastu-Kapur S., Nguyen M., Raha D., Ermini F., Haditsch U., Araujo J., De Lannoy I., Ryder M.I., Dominy S.S., Lynch C. (2020). Treatment of *Porphyromonas gulae* infection and downstream pathology in the aged dog by lysine-gingipain inhibitor COR388. Pharmacol. Res. Perspect..

[53] Chen C., Hemme C., Beleno J., Shi Z.J., Ning D., Qin Y., Tu Q., Jorgensen M., He Z., Wu L. (2018). Oral microbiota of periodontal health and disease and their changes after nonsurgical periodontal therapy. ISME J..

[54] Bathini P., Foucras S., Dupanloup I., Imeri H., Perna A., Berruex J.L., Doucey M.A., Annoni J.M., Auber Alberi L. (2020). Classifying dementia progression using microbial profiling of saliva. Alzheimers Dement..

[55] Liu X.X., Jiao B., Liao X.X., Guo L.N., Yuan Z.H., Wang X., Xiao X.W., Zhang X.Y., Tang B.S., Shen L. (2019). Analysis of Salivary Microbiome in Patients with Alzheimer’s Disease. J. Alzheimers Dis..

[56] Muñana K.R., Jacob M.E., Callahan B.J. (2020). Evaluation of fecal Lactobacillus populations in dogs with idiopathic epilepsy: A pilot study. Anim. Microbiome..

[57] Coman M.M., Verdenelli M.C., Cecchini C., Belà B., Gramenzi A., Orpianesi C., Cresci A., Silvi S. (2019). Probiotic characterization of Lactobacillus isolates from canine faeces. J. Appl. Microbiol..

[58] Lee H.J., Cho J.H., Cho W.J., Gang S.H., Park S.H., Jung B.J., Kim H.B., Song K.H. (2022). Effects of Synbiotic Preparation Containing Lactobacillus gasseri BNR17 on Body Fat in Obese Dogs: A Pilot Study. Animals.

[59] Nakagawa H., Shiozaki T., Kobatake E., Hosoya T., Moriya T., Sakai F., Taru H., Miyazaki T. (2016). Effects and mechanisms of prolongevity induced by Lactobacillus gasseri SBT2055 in Caenorhabditis elegans. Aging Cell.

[60] Yun S.W., Kim J.K., Lee K.E., Oh Y.J., Choi H.J., Han M.J., Kim D.H. (2020). A Probiotic Lactobacillus gasseri Alleviates Escherichia coli-Induced Cognitive Impairment and Depression in Mice by Regulating IL-1β Expression and Gut Microbiota. Nutrients.

[61] Liu S., Wang Y., Zhao L., Sun X., Feng Q. (2020). Microbiome succession with increasing age in three oral sites. Aging.

[62] EHead N.W.M., Cotman C.W., Hof P.R., Mobbs C.V. (2001). Neurobiological models of aging in the dog and other vertebrate species. Functional Neurobiology of Aging.

[63] Wallis C., Holcombe L.J. (2020). A review of the frequency and impact of periodontal disease in dogs. J. Sm. Anim. Pract..

